# Influence of riders’ physical activity on improving seat balance in the context of horse welfare – A pilot study

**DOI:** 10.1371/journal.pone.0344969

**Published:** 2026-04-15

**Authors:** Paulina Zeliszewska-Duk, Anna Skowerska-Wiśniewska, Izabela Wilk, Beata Nowicka, Mariusz Duk, Katarzyna Strzelec

**Affiliations:** 1 Department of Horse Breeding and Use, Faculty of Animal Breeding and Bioeconomy, University of Life Sciences in Lublin, Lublin, Poland; 2 Department and Clinic of Animal Surgery, Faculty of Veterinary Medicine, University of Life Sciences in Lublin, Lublin, Poland; 3 Department of Electronics and Information Technology, Faculty of Electrical Engineering and Computer Science, Lublin University of Technology, Lublin, Poland; Public Library of Science, UNITED KINGDOM OF GREAT BRITAIN AND NORTHERN IRELAND

## Abstract

This study aimed to investigate the impact of gym exercises for riders on their posture and the subsequent effect on reducing discomfort for their horses during training, using electronic pressure sensors. The study involved 20 warmblood horses aged 5–10 years, regularly ridden under saddle, and four riders of similar height and body weight. The study group was divided into a control group (10 horses) and an experimental group (10 horses). The horses were trained 5 days a week for 60 days in total. Riders trained three times a week for approximately 60 minutes. The exercises were designed to improve the riders’ flexibility, mobility, and fitness. A pad with electronic pressure sensors was placed beneath the saddle to detect pressure force and changes in the rider’s position. The results clearly indicate that exercise has a positive impact on riders’ balance maintenance. In each analysed case, the experimental group observed a reduction in amplitude non-uniformity in the measurement performed upon completion of riders’ training. This method may help more easily assess seat irregularities and correct them through appropriately selected exercises. Improving the rider’s balance should significantly improve the horse’s comfort while riding.

## Introduction

The welfare of horses used for riding under saddle is an extremely important issue that is receiving increasing attention [1]. Since these horses are exposed to a variety of dangers, primarily injuries and diseases, it is essential to provide them with suitable living conditions and care to ensure their welfare. Currently, the main use of the horses is horseback riding [2]. Across the world, various activities related to horse riding are available, ranging from recreational riding to equestrian sports and racing. In any case, the rider’s body weight and balance affect the horse’s back. A lack of balance, or even minor mistakes in the rider’s seating, will have an adverse effect on the muscles, causing discomfort and, consequently, muscle tension or even lameness [3].

Research focused on improving horse comfort while riding primarily examines the correct fitting of equipment, especially the saddle, the use of various materials in the pads, and subjective assessment of the effect of the rider’s seating on the horse’s motor skills [4]. The method commonly used to assess equipment fit is thermal imaging, which enables the identification of incorrect weight distribution while riding or an incorrectly fitted saddle [5–7]. Horses indicate they are in pain through various expressions, such as ears that are “low” or asymmetrical, sharp-looking eyes, a withdrawn or tense gaze, flared nostrils, and tension in the lips, chin, and specific face muscles [8]. However, an improperly fitted saddle also affects the rider’s seating. An improperly sized or shaped saddle can cause back pain, hip pain, sores under the ‘seating bones’, and perineal injuries [9].

Back pain in horses is a common problem that can arise from various causes, including the rider’s impact [10]. Muscle flexibility, balance and the rider’s skills have a huge effect on how the horse feels the weight and pressure on its back. Incorrect riding technique and a lack of skill on the part of the rider can lead to strain, injuries and, consequently, back pain in the horse. Even a slight shift of the rider’s body weight means that the horse has to regain its balance. If a horse has to constantly change its balance to compensate for the rider’s mistakes, it is more difficult for it to move efficiently and freely [11,12].

The correct rider’s seating and balance are essential for the horse’s back health [13]. Improving the rider’s seating requires time, patience, and commitment. Gym-based exercise can be a valuable supplement to riding training, helping strengthen the muscles responsible for proper posture and balance in the saddle [14]. Exercising at a gym, which strengthens muscles and improves balance, actually translates into better rider seating [15,16]. Better seating means the rider sits more stably in the saddle and distributes their body weight evenly, reducing pressure on the horse’s back and enhancing its comfort during the ride.

It was hypothesised that additional physical activity of riders would have a positive effect during horse riding. The study aimed to confirm whether gym exercises for riders improve their posture and reduce discomfort for their horses during training using electronic pressure sensors.

## Materials and methods

The study was conducted according to the guidelines of the Declaration of Helsinki. Ethical review and approval were waived, due to non-invasiveness of the study. The horses were subjected to non-invasive procedures in view of European directive 2010/63/EU and Polish laws related to ethics in animal experimentation. The horses belonged to the University of Life Sciences in Lublin, Poland and were maintained in a riding center under the care of one of the authors who monitored their welfare and veterinary state as assigned by the university. The procedures took place in a familiar environment that they experienced daily and did not cause them any pain, suffering, or damage. The horses were clinically healthy, with any signs of sense disorders, no clinical signs of lameness or musculoskeletal injury; they demonstrated a comparable condition and athletic ability. All procedures were conducted in accordance with the Polish Animal Protection Act (21 August 1997).

### Experiment design

The study involved 20 warmblood horses aged 5–10 years, regularly ridden under saddle, and four riders of similar height and body weight. The study group was divided into a control group (10 horses) and an experimental group (10 horses). Each rider was assigned five randomly selected horses.

The horses were used for riding 6 days a week for approximately 1–2 hours per day and were kept in a box stall stable at a single riding centre. They also had access to open-air runs for 4–6 hours per day.

The experiment lasted 60 days. The first measurement using electronic pressure sensors was conducted on the first day of the experiment for the control group, and the next day for the experimental group. Both groups participated in standardised dressage training every other day. Additionally, the experimental group riders participated in regular general development training at the gym with a personal trainer. The last sensor measurement was conducted 60 days after the first measurement for both the control and experimental groups.

The horses were trained 5 days a week for 60 days in total. A pad containing electronic pressure sensors that reacted to the force of pressure and changes in the rider’s position in the saddle was placed under the saddle. Training sessions for the horses were held in a roof-covered manège, or riding arena, measuring 75 x 36 m, on a specialised riding surface.

Each training session included:

- 10 minutes of walk on a loose rein, with a change of direction every two laps,
- 10 minutes of posting trot, with a change of direction every two laps,
- one lap of the hall at a walk to the left, and one to the right,
- sitting trot to the left, running a 20 m circle at both ends of the hall, followed by a change in direction diagonally, and performing the above-mentioned exercises to the right,
- one lap of the hall at a walk to the left, and one to the right,
- sitting trot and trot-to-canter transition starting from the left leg, running a 20 m circle at both ends of the hall, followed by a change in direction diagonally with a transition to trot at X, and performing the above-mentioned exercises to the right,
- one lap of the hall at posting trot to the right, and one to the left,
- 10 minutes of walk on a loose rein, with a change of direction every two laps.

The study involved four riders with riding skills at least equivalent to those required for the Silver Horse Riding Badge of the Polish Equestrian Federation, who were randomly assigned to the control and experimental groups. The riders in the experimental group underwent regular functional training at the gym, aimed at improving motor coordination, specifically balance and flexibility, which significantly impact the quality of the rider’s seating. A personal trainer combined the exercises into appropriate sets based on each rider’s needs. At that time, the control group performed no additional activities.

The following gym exercises were used:

- Plank: Strengthens core muscles, improves spine and pelvic stability.
- Squats: Strengthen the muscles of the legs and buttocks, improve balance and stability.
- Deadlift: Strengthens the muscles of the back, buttocks and legs, improves strength and stability.
- Dumbbell rowing: Strengthens the muscles of the back and arms, improves posture.
- Exercises with an exercise ball: Improve balance and activate deep muscles.
- Stretching: Improves flexibility and increases the range of motion.

Riders trained three times a week (Mondays, Wednesdays, and Fridays). Each session lasted approximately 60 minutes.

Tests were conducted in both the experimental and control groups, utilising an electronic system to record the rider’s seating. The tests were conducted in two rounds. The first test round was conducted identically in both groups, i.e., without prior preparation of the horses or riders. The second round of tests was conducted 60 days later. The recordings for all the riders and horses in both rounds were performed at three different gaits (posting trot, sitting trot, canter), and in different directions of the horse’s motion (straight ahead, turning to the right and turning to the left). Due to the large volume of data and the fact that the conclusions drawn from the results obtained are identical for different gaits and different directions of motion, the basic statistical parameters are presented in tabular form in the following part of the study only for the posting trot in a straight-ahead motion.

### The structure and principle of operation of the seating monitoring system

The rider’s seating monitoring system was used to determine whether the seating was correct. To this end, airbags were placed between the saddle and the horse’s back. For research purposes, a corrective saddle pad from Winderen was used, whose design properties enabled easy placement of airbags inside it.

**Fig 1:**
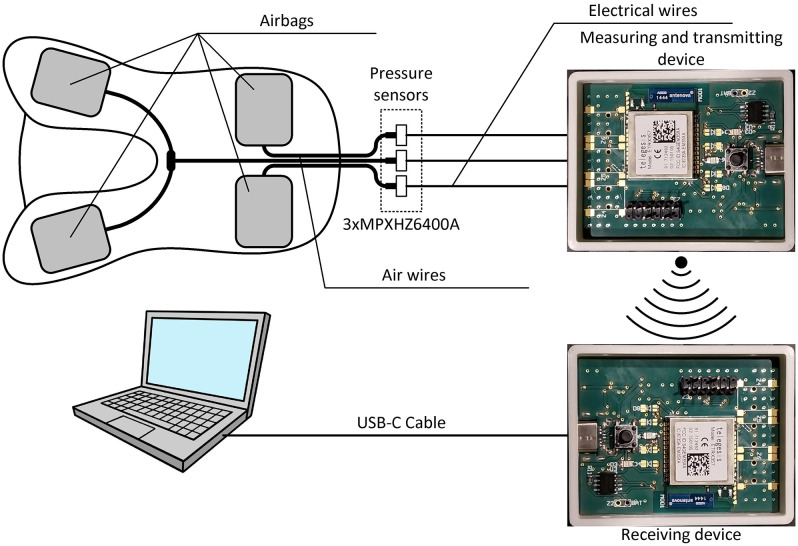
The structure of the electronic rider’s seating control system.

Placing airbags between the inner layers of the pad ensured that the horse participating in the study felt no difference between the everyday work and work during data collection. Four airbags were placed in the pad, of which two operated independently, and two were combined into a pair (with identical pressure in both airbags). The airbag pair was placed under the front of the saddle, allowing an analysis of changes in the rider’s seating impact on the pommel. Additionally, separate airbags were placed under the rear part of the saddle, facilitating the investigation of the rider’s seating impact on the cantle. These airbags were also divided into left and right sides, enabling the detection of balance problems or asymmetries resulting from the horse’s conformation faults. The arrangement of the airbags is shown in Fig 1.

During system preparation for testing, the pad was placed in a 20 mm-high pocket, and all airbags were then filled to the same initial pressure (100 kPa) to ensure repeatable measurements during testing. The pressure readings from the seating monitoring system were calibrated and confirmed using an LLS-01 laboratory fibre-optic pressure sensor from SYLEX.

Changes in rider’s seating altered the airbag pressure. The air was vented from the airbags outside the saddle via thin pneumatic tubes, then fed to micromechanical piezoresistive semiconductor pressure sensors (MPXHZ6400A type, Freescale Semiconductor). The above pressure sensors in the system served as transducers, converting a physical quantity, namely pressure, into an electrical value in the form of an output voltage. The voltage signals from the three pressure sensors (one for the front airbag and two for the rear airbags) were transmitted through cables to the measuring and transmitting system. There, the signals were converted to digital form and sent via a radio link to the receiving system, which was connected to a computer. The system’s structure is illustrated in Fig 1.

The measuring and transmitting system was built using a Texas Instruments MSP430F5529 microcontroller. This type of microcontroller was used due to its high performance-to-power consumption ratio (high performance/ low power consumption). In addition, the above microcontroller was equipped with two UART interfaces. One of these was used for direct connection to the computer for unit configuration, while the other was used for transmission via a wireless transmission module. The microcontroller also had a built-in 12-bit analogue-to-digital converter that enabled measurements of voltages originating from pressure sensors.

Wireless transmission was performed using the ETRX357 module from Silicon Labs, which enabled communication with ZigBee. The application of this transmission system enabled the establishment of a wireless network, allowing simultaneous data recording from multiple measuring and transmitting devices. The range of wireless transmission between two points was at least 100 m. In the ZigBee network, it was possible to extend the range by relaying transmissions between modules (e.g., when the distance between the transmitter and the receiver was too great, an additional transmitter acted as a retransmission element).

The measuring and transmitting system was equipped with a 2000 mAh lithium-ion battery and a charging management system. This battery capacity enabled approximately 10 hours of continuous operation. The battery was charged using a charger equipped with a USB output.

**Fig 2:**
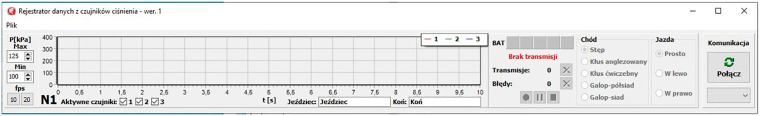
Computer application window for data reception and acquisition.

For cost optimisation reasons, the receiving system had the same design as the measuring and transmitting system. No voltage-signal-measuring inputs were used, and it was devoid of a battery and a charging management system. The main difference between these two systems was the microcontroller software. The task of the receiving system was to receive data wirelessly from the measuring and transmitting systems and to send it via a USB drive to the computer, where a dedicated application enabled real-time viewing and acquisition of the transmitted data. The application window is shown in Fig 2.

To verify the system’s correct operation, preliminary tests were conducted that involved recording the time series of changes in airbag pressure. The measurements were taken at a frequency of 20 Hz.

### Statistical methods

The values of individual motion parameter measurements were compiled in an Excel spreadsheet database. The statistical analysis was carried out using the Statistica program. In the first step, the data were tested for normality. The Kolmogorov-Smirnov, Lilliefors, and Shapiro-Wilk tests indicated that the tested values followed a normal distribution. In the next step, an ANOVA for independent groups was performed, with the subsequent measurement as the factor. The significance of the differences was determined using Tukey’s t-test. Moreover, descriptive statistics were computed, including means, medians, maximum and minimum values, and standard deviations for the studied parameters. The amplitudes and amplitude non-uniformities were also calculated.

## Results

Table 1 presents the results of multivariate significance tests.

**Table 1:** Parameter values for ANOVA analysis.

Effect	Value	F	Effect (df)	Error (df)	p
Free expression	0.061875	208.4727	4	55	0.000000
Subsequent measurement	0.073183	1.9970	4	55	0.007745

The Table 2 shows the results for the recorded time series of individual riders for the first and second repetitions of the study. In the first measurement, all riders showed irregularity when comparing the loads on the right and left sides. The results also indicate that for riders 1 and 2, a noticeable change occurred in the second measurement. In both cases, greater regularity was observed when the load was applied to the right and left sides.

**Table 2:** The results for the recorded time series of individual riders in the first and second stages of the study.

Rider	Round	Airbag	M [kPa]	SD
**1** **experimental group**	1	Left	117.00a	0.60
		Right	114.64a	0.57
	2	Left	107.91b	0.59
		Right	107.75b	0.57
**2** **experimental group**	1	Left	116.74a	0.66
		Right	126.98a	1.25
	2	Left	117.08b	1.00
		Right	115.94b	1.24
**3** **control group**	1	Left	118.05a	0.68
		Right	123.14a	1.19
	2	Left	116.05a	0.68
		Right	124.14a	0.57
**4** **control group**	1	Left	116.09a	1.94
		Right	120.73a	1.74
	2	Left	114.73a	0.64
		Right	120.59a	0.71

The means marked with different lowercase (a, b) are significantly different at α = 0.05.

When analysing the time series recordings at posting trot for riders in the experimental group, it was evident that, in each element, the non-uniformity of pressure amplitude changed during the second measurement (see Table 3). The highest values of amplitude non-uniformity, reaching approximately 24%, were noted when riding straight ahead. A similar situation was also observed when turning right and left. In each case, a decrease in the amplitude non-uniformity by at least several per cent was observed.

**Table 3:** Statistical parameters of the recorded time series at posting trot.

Direction	Round	Airbag	M	Me	σ	Min	Max	A	ΔA
kPa	kPa	–	kPa	kPa	kPa	%
**Straight ahead**	1	Left	110.44	107.80	6.28	103.00	124.90	21.90	31.75
		Front	111.98	110.60	3.94	107.10	120.60	13.50	
		Right	109.13	107.40	4.37	104.20	120.10	15.90	
	2	Left	108.34	106.46	4.41	103.10	118.43	15.33	6.88
		Front	111.66	110.54	3.47	107.30	119.54	12.24	
		Right	108.24	106.66	4.00	103.78	118.09	14.31	
**Left turn**	1	Left	111.69	109.20	5.53	106.10	128.00	21.90	15.23
		Front	111.73	111.20	3.28	107.50	119.70	12.20	
		Right	109.44	107.80	4.83	102.70	121.50	18.80	
	2	Left	111.39	109.25	4.66	105.97	122.60	16.63	0.98
		Front	111.02	110.50	2.88	107.35	117.61	10.26	
		Right	109.05	107.49	4.49	102.63	119.10	16.47	
**Right turn**	1	Left	111.58	109.40	5.67	104.50	125.20	20.70	16.75
		Front	111.19	110.60	2.96	107.30	117.70	10.40	
		Right	110.73	109.30	4.69	105.20	122.70	17.50	
	2	Left	110.03	108.41	4.42	104.51	120.66	16.15	1.35
		Front	110.96	110.41	2.70	107.17	116.53	9.36	
		Right	110.27	108.97	4.29	105.10	121.03	15.93	

Value designations in the tables:

M – mean value,

Me – median,

σ – standard deviation,

Min – minimum value,

Max – maximum value,

A – amplitude (a difference between the values A = Max – Min),

ΔA – amplitude non-uniformity (ΔA=(AL–AP)/Aśr – where: AL – amplitude of the left side, AP –amplitude of the right side, Aśr – mean amplitude of the left and right sides).

**Fig 3:**
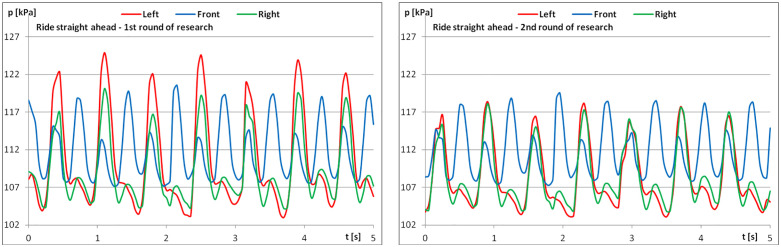
The course of pressure changes in the airbags at posting trot – riding straight ahead (a) before starting the exercises; (b) after finishing the exercises.

**Fig 4:**
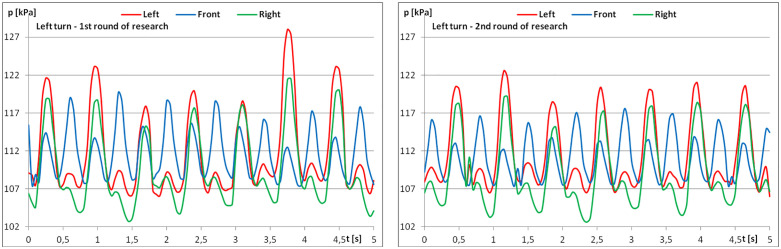
The course of pressure changes in the airbags at posting trot – riding to the left (a) before starting the exercises; (b) after finishing the exercises.

**Fig 5:**
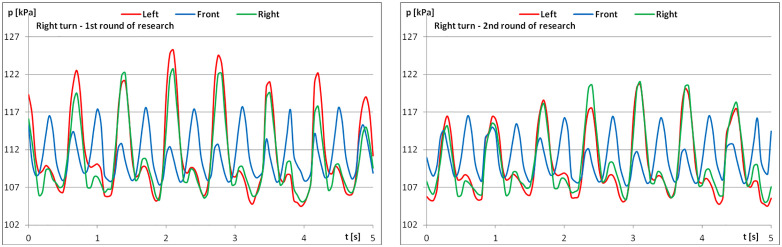
The course of pressure changes in the airbags at posting trot – riding to the right (a) before starting the exercises; (b) after finishing the exercises.

The diagrams show examples of pressure force recordings for one of the riders under study (Figs 3–5). In each analysed part, an improvement in pressure regularity was evident during the second repetition.

Regarding measurements of the sitting trot of riders in the experimental group, a decrease in the non-uniformity of the pressure amplitude during the second repetition is again observed. These were particularly evident when turning left, where they accounted for over 10% (see Table 4).

**Table 4:** Statistical parameters of the recorded time series at sitting trot.

Direction	Round	Airbag	M	Me	σ	Min	Max	A	ΔA
			kPa	kPa	–	kPa	kPa	kPa	%
**Straight ahead**	1	Left	115.00	114.80	5.28	106.60	124.50	17.90	9.36
		Front	109.70	109.00	3.18	105.80	115.50	9.70	
		Right	112.27	112.30	4.65	105.10	121.40	16.30	
	2	Left	113.70	113.14	4.14	106.58	121.36	14.77	0.70
		Front	109.50	108.62	2.89	105.74	114.65	8.91	
		Right	112.33	112.19	4.14	105.89	120.56	14.67	
**Left turn**	1	Left	115.92	115.10	5.52	107.00	125.70	18.70	16.81
		Front	109.89	108.80	3.00	106.00	115.80	9.80	
		Right	112.08	111.70	4.47	105.20	121.00	15.80	
	2	Left	115.04	114.95	4.24	106.85	121.85	15.00	5.34
		Front	109.76	109.61	2.69	106.10	114.92	8.82	
		Right	113.45	113.27	4.00	106.88	121.10	14.22	
**Right turn**	1	Left	114.91	115.10	4.83	107.50	124.80	17.30	4.13
		Front	110.04	109.00	3.13	105.80	115.70	9.90	
		Right	112.46	112.30	4.24	105.00	121.60	16.60	
	2	Left	112.96	113.59	4.14	105.22	119.80	14.58	1.94
		Front	109.36	108.64	2.85	105.31	114.13	8.82	
		Right	112.12	112.20	3.87	105.00	119.30	14.30	

Value designations in the tables:

M – mean value,

Me – median,

σ – standard deviation,

Min – minimum value,

Max – maximum value,

A – amplitude (a difference between the values A = Max – Min),

ΔA – amplitude non-uniformity (ΔA=(AL–AP)/Aśr – where: AL – amplitude of the left side, AP – amplitude of the right side, Aśr – mean amplitude of the left and right sides).

**Fig 6:**
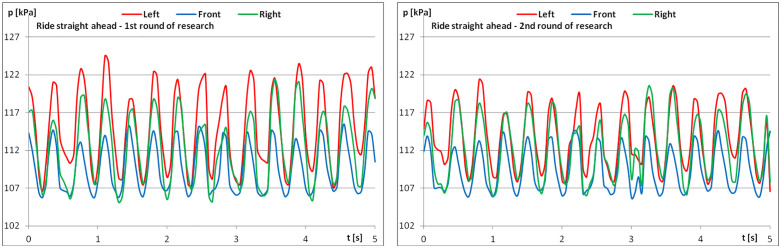
The course of pressure changes in the airbags at sitting trot – riding straight ahead (a) before starting the exercises; (b) after finishing the exercises.

**Fig 7:**
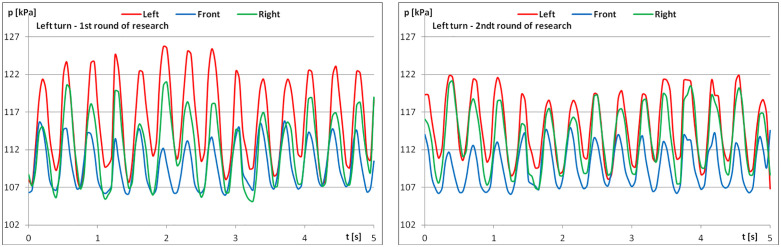
The course of pressure changes in the airbags at sitting trot – riding to the left (a) before starting the exercises; (b) after finishing the exercises.

**Fig 8:**
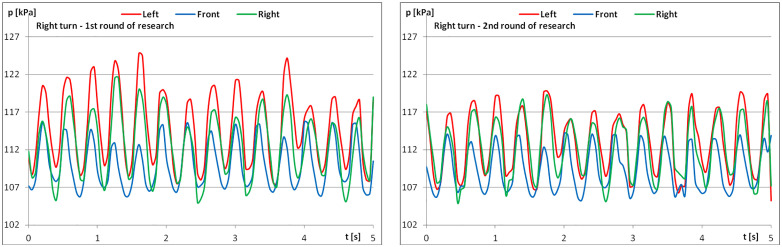
The course of pressure changes in the airbags at sitting trot – riding to the right (a) before starting the exercises; (b) after finishing the exercises.

Figs 6–8 show examples of recordings for one rider operating at a sitting trot. Again, the recordings from the second repetition were more regular.

The next part of the experiment was to record a time series at canter (see Table 5). During the measurements, a decrease in the amplitude non-uniformity values was again observed during the second repetition for riders in the experimental group. The greatest differences were noted when turning right. During the recorded measurements, the lowest amplitude non-uniformity differences were observed during left turns.

**Table 5:** Statistical parameters of the recorded time series at canter.

Direction	Round	Airbag	M	Me	σ	Min	Max	A	ΔA
			kPa	kPa	–	kPa	kPa	kPa	%
**Straight ahead**	1	Left	115.49	116.10	6.15	106.00	127.00	21.00	25.81
		Front	108.13	108.40	1.91	104.60	111.80	7.20	
		Right	112.94	112.90	4.85	105.80	122.00	16.20	
	2	Left	112.85	113.05	4.70	105.63	120.70	15.08	1.5
		Front	107.54	107.86	1.78	104.44	110.92	6.48	
		Right	112.06	111.85	4.52	105.55	120.40	14.85	
**Left turn**	1	Left	115.22	115.30	5.75	106.60	125.40	18.80	10.06
		Front	108.28	108.70	1.93	104.60	112.80	8.20	
		Right	112.29	112.40	5.38	104.60	121.60	17.00	
	2	Left	112.67	112.78	4.42	105.35	120.35	15.00	0.40
		Front	107.88	108.31	1.74	104.44	111.82	7.38	
		Right	111.98	112.48	4.85	105.10	120.04	14.94	
**Right turn**	1	Left	116.63	117.00	6.32	107.20	128.80	21.60	42.02
		Front	108.35	108.80	1.89	105.30	111.70	6.40	
		Right	111.89	111.90	3.93	105.60	119.70	14.10	
	2	Left	111.30	111.42	4.27	105.05	118.18	13.13	6.28
		Front	107.75	108.31	1.69	104.80	110.83	6.03	
		Right	111.14	111.22	3.62	105.64	117.97	12.33	

Value designations in the tables:

M – mean value,

Me – median,

σ – standard deviation,

Min – minimum value,

Max – maximum value,

A – amplitude (a difference between the values A = Max – Min),

ΔA – amplitude non-uniformity (ΔA=(AL–AP)/Aśr – where: AL – amplitude of the left side, AP – amplitude of the right side, Aśr – mean amplitude of the left and right sides).

**Fig 9:**
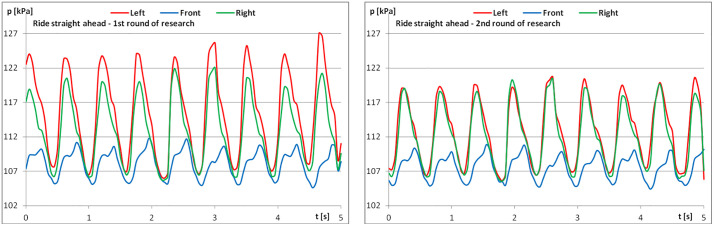
The course of pressure changes in the airbags at canter – riding straight ahead (a) before starting the exercises; (b) after finishing the exercises.

**Fig 10:**
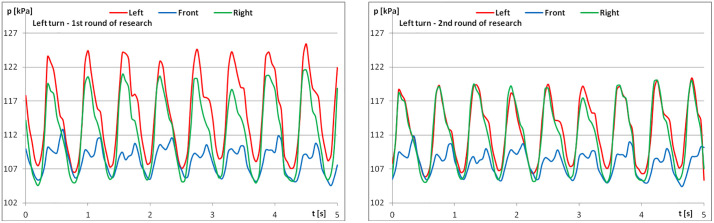
The course of pressure changes in the airbags at canter – riding to the left (a) before starting the exercises; (b) after finishing the exercises.

**Fig 11:**
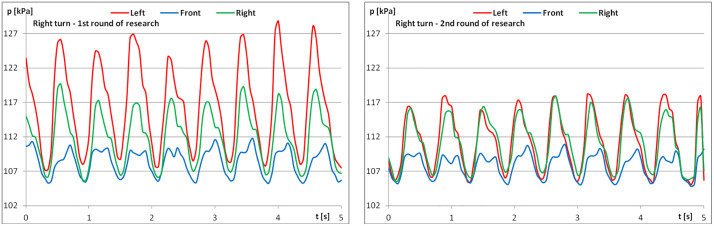
The course of pressure changes in the airbags at canter – riding to the right (a) before starting the exercises; (b) after finishing the exercises.

When analysing pressure change graphs for the example rider, the change in regularity for the first and second measurements is visible (Figs 9–11). Greater regularity can be observed during the second measurement, i.e., after the riders performed the recommended series of exercises.

## Discussion

Modern biomechanics increasingly employs mobile technologies and motion analysis systems in real-world, unconstrained laboratory conditions. This allows recording a horse’s natural behaviour and movement during training or competition, bringing scientific analysis closer to practice. The use of inertial sensors mounted on the rider’s limbs, back, or helmet enables real-time tracking of changes in balance and movement dynamics, which is useful for both sports training and post-injury rehabilitation assessment [17–19].

The present study aimed to investigate the contribution of gym exercise to the improvement of riders’ physical fitness and muscle flexibility, and, consequently, to their ability to better cooperate with horses using proper seating. A system of airbags and sensors, specifically designed for this experiment, was used as a measurement method, enabling the determination of the force of the riders’ pressure at the front and on the right and left sides of the saddle. The technique used in the current study differs significantly from those used by other authors to measure pressure. [20]. During the experiment, a pad with mounted air bags was used, while other authors primarily used pressure mats [21,22]. This technique could be an innovative and simple solution, enabling its use on a wider scale.

When analysing recordings from the control and experimental groups, a distinct difference was observed. In the control group, differences in load between the right and left sides were observed at the first and last measurements, suggesting asymmetry. Variations in the pressure distributed between the sides can consequently lead to the horse’s discomfort while riding. When the rider does not maintain their balance, it significantly affects the mechanics of the horse’s motion and leads to disturbances in motion [23–25]. Long-term impact of an irregular force on the horse’s back will lead to muscle tension and additionally make it harder for the rider to control the horse. It is also worth noting that the study involved riders with experience and skills that allowed them to compete in class P events in both dressage and show jumping. The observed differences were clearly visible in their case, whereas riding horses is largely recreational, used by riders with much less experience and lower seating flexibility. On the other hand, certain tendencies can be observed, but each body will react to training individually. Previous studies examining the effects of rider asymmetry on horses have used video recordings, pressure-sensing saddle mats, and accelerometers to assess the rider’s influence on the horse’s thoracolumbar spine range of motion and gait quality. Rider-induced changes in horse movement can impact the horse’s overall movement experience, athletic performance, and even long-term performance [26]. The study clearly shows that choosing appropriate exercises will positively affect the riders’ balance and, thus, reduce the irregularity of the forces acting on the horse’s back. The presented results evidently indicated a lack of symmetry, regardless of the manner in which the horse was ridden. This is particularly important in terms of the forces acting on the horse. According to De Cocq et al. [11], horses with a more elastic gait are subjected to greater inertial and gravitational loads imposed by the rider.

Another aspect was the use of the meters themselves and data analysis. In this paper, the authors focused mainly on interpreting the results for riders in the experimental group. Moreover, results for riders across all gaits under study were presented. The system itself proved exceptionally accurate, and analysis of the recordings enabled the diagnosis of problems with individual riders’ seating and posture. The results indicated that a modified pad under the saddle can be a valuable tool for identifying seating irregularities, but it should be used on an individual basis, targeting a specific rider.

## Conclusions

The results clearly indicate that exercise has a positive impact on riders’ balance maintenance. In each case, the experimental group observed a reduction in amplitude non-uniformity in the second measurement. This method may help more easily assess seat irregularities and correct them through appropriately selected exercises. Improving the rider’s balance should significantly improve the horse’s comfort while riding.
